# Ste20‐like kinase activity promotes meiotic resumption and spindle microtubule stability in mouse oocytes

**DOI:** 10.1111/cpr.13391

**Published:** 2022-12-29

**Authors:** Ke Song, Xiuying Jiang, Xiangning Xu, Ye Chen, Jiaqi Zhang, Ying Tian, Qian Wang, Jing Weng, Yuanjing Liang, Wei Ma

**Affiliations:** ^1^ Department of Histology and Embryology School of Basic Medical Sciences, Capital Medical University Beijing China; ^2^ Division of Sport Anatomy School of Sport Science, Beijing Sport University Beijing China

## Abstract

Ste20‐like kinase (SLK) is involved in cell proliferation and migration in somatic cells. This study aims to explore SLK expression and function in mouse oocyte meiosis. Western blot, immunofluorescence, Co‐immunoprecipitation, drug treatment, cRNA construct and in vitro transcription, microinjection of morpholino oilgo (MO) and cRNA were performed in oocytes. High and stable protein expression of SLK was detected in mouse oocyte meiosis, with dynamic distribution in the nucleus, chromosomes and spindle apparatus. SLK phosphorylation emerges around meiotic resumption and reaches a peak during metaphase I (MI) and metaphase II. SLK knockdown with MO or expression of kinase‐dead SLK K63R dramatically delays meiotic resumption due to sequentially suppressed phosphorylation of Polo‐like kinase 1 (Plk1) and cell division cycle 25C (CDC25C) and dephosphorylation of cyclin‐dependent kinase 1 (CDK1). SLK depletion promotes ubiquitination‐mediated degradation of paxillin, an antagonist to α‐tubulin deacetylation, and thus destroys spindle assembly and chromosome alignment; these phenotypes can be substantially rescued by exogenous expression of SLK kinase active fragment. Additionally, exogenous SLK effectively promotes meiotic progression and spindle assembly in aging oocytes with reduced SLK. Collectively, this study reveals SLK is required for meiotic resumption and spindle assembly in mouse oocyte meiosis.

## Graphical abstract

Around meiotic resumption in oocytes, SLK inspires a signal cascade involving Plk1, CDC25C and CDK1, therefrom bringing about metaphase‐promoting factor maturation, and during the following meiotic progression, promotes microtubule acetylation by regulating paxillin stability to ensure correct spindle assembly and chromosome alignment.

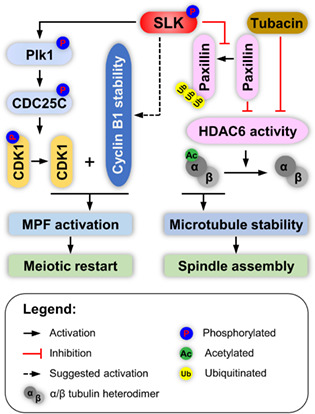

## 1 INTRODUCTION

Mammalian oocytes of high‐developmental‐competence are required for successful fertilization and subsequent embryo development, which initiates a new life.^1^ Fully‐grown oocytes are arrested at the diplotene stage of the first meiotic prophase, known as the germinal vesicle (GV) stage.^2^ Following the ovulatory luteinizing hormone (LH) surge, the resumption of meiosis occurs with the signature of germinal vesicle breakdown (GVBD) followed by chromatin condensation.^3^, ^4^ During the prometaphase of meiosis I (Pro‐MI), the acentrosomal spindle is assembled along with the congression of condensed chromosomes.^5^ Upon all chromosomes are correctly aligned and stably attached by microtubules from spindle poles at metaphase I (MI), the meiotic cell cycle moves to anaphase I (AI), during which the homologous chromosomes are segregated, and the first polar body (PB1) is discharged.^6^ The orderly meiotic progression is essential for the high‐quality oocytes and, furthermore, female reproductive health.^1^, ^7^ The abnormal oocyte meiotic process leads to embryo aneuploidy, a significant cause of infertility, abortion and fetal deformity.^7^

The fully‐grown oocytes accumulate large quantities of mRNAs and proteins and stop de novo mRNA transcription at the GV stage, so the meiotic progression is regulated by a complex and cascading network involving multiple proteins and signalling molecules.^8^, ^9^, ^10^ Concretely, meiotic resumption and spindle assembly is promoted by orchestrated post‐translational modification of proteins, particularly phosphorylation or dephosphorylation catalysed by protein kinases or phosphatases.^11^ The full activation of metaphase‐promoting factor (MPF) is required for meiotic resumption, which is combined of regulatory subunit cyclin B and catalytic subunit cyclin‐dependent kinase 1 (CDK1). CDK1 dephosphorylation is critical to sparking MPF activity, stimulated by CDC25C, a phosphatase activated by the upstream sequence of phosphorylation reactions.^12^ In mammalian oocytes, the spindle assembly is an acentrosomal process controlled by the unique microtubule organizing centre (MTOC). Previous evidence illustrates some kinases are recruited to MTOC around GVBD, such as (PKC), and polo‐like kinase 1 (Plk1), promoting the morphological and functional maturation of MTOC through phosphorylating specific substrates. Spindle pulling force on chromosomes depends on the dynamic assembly and reasonable stability of microtubules, which is coordinately regulated by multiple post‐translational modifications of tubulin. As has been reported, α‐tubulin Thr349 phosphorylation affects microtubule assembly, while its acetylation on Lys40 promotes microtubule stability, and CDK1‐induced β‐tubulin phosphorylation on Ser172 inhibits tubulin incorporation into microtubules from metaphase to telophase in somatic mitosis.^13^, ^14^, ^15^ Despite the substantial investigation, it is still not fully understood the enzymatic regulation mechanism governing the dynamic post‐translational modification of tubulin in spindle assembly during oocyte meiotic division.

Ste20‐like kinase (SLK) belongs to the Ste20 family of serine/threonine protein kinases, which is ubiquitously expressed in tissues and cell lines,^16^ and its depletion leads to embryonic lethality.^17^ Multiple phosphorylation sites of SLK have been identified, and Thr183, Thr193 and Ser189 have been shown to be essential for SLK activity.^18^, ^19^ SLK roles have been identified in diverse biological processes, such as cell cycle control, apoptosis, cytoskeletal dynamic and cell migration. It has been reported that depletion of SLK results in cell cycle arrest in the early G2 in fibroblast cells, which is associated with the abated phosphorylation and activation of Plk1, the early trigger for G2/M transition.^20^, ^21^ SLK knockdown or expression of kinase‐dead SLK variant destroys microtubules organizing in interphase fibroblasts, resulting from the decreased p150 (Glued) capacity in regulating centrosome anchoring by forming a complex with NuMA.^22^ In vascular smooth muscle cells, SLK promotes the phosphorylation of skeleton protein RhoA on Ser188 and thus limits its activity in cell contraction,^23^, ^24^ while in lung airway smooth muscles, SLK can phosphorylate Plk1 on Thr210 with the participation of acetylcholine (Ach), and sequent phosphorylation of vimentin and paxillin, which induces cell contraction.^25^ So SLK's function in regulating cell cycle and cytoskeleton dynamics is dependent on its phosphorylation activity on specific substrate molecules.

In this study, our results show that SLK activity promotes the meiotic resumption in mouse oocytes by inspiring a signal cascade comprised of Plk1, CDC25C and CDK1 and boosts the acetylation and stability of spindle microtubule by sustaining paxillin level against ubiquitination‐mediated degradation. SLK is reduced in aging ovaries and oocytes, and the exogenous SLK active fragment can ameliorate aging‐associated impaired meiotic progression and spindle morphology in oocytes.

## 2 MATERIALS AND METHODS

### 2.1 Oocyte collection and in vitro culture

All the animal experiments were strictly conducted following the policies and instructions of the Care and Use of Animals in Research and Teaching and approved by the Animal Care and Use Committee of Capital Medical University with the approval No AEEI‐2020‐151. To collect fully‐grown GV oocytes, 3‐week‐old CB6F1 (C57BL/6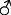 × BALB/C
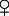
F1) female mice were injected with 5 IU pregnant mare serum gonadotropin (PMSG) (Ningbo Second Hormone Factory). After 44–48 h, cumulus‐oocyte complexes (COCs) were isolated from the ovaries and incubated in minimal essential medium (MEM) containing 3 mg/ml bovine serum albumin (BSA, Sigma) and 10% fetal bovine serum (FBS, Gibco) at 37°C in 5% CO_2_ atmosphere. At different time points after culture, oocytes were collected for subsequent analysis.

### 2.2 Microinjection and morpholino oligo interference

Fully‐grown GV oocytes were micro‐injected with 10–15 pl 1 mM control (5′‐CCTCTTACCTCAGTTACAATTTATA‐3′) or *Slk* morpholino oligo (5′‐ACATTTTTCCAAGCCCAGCAGAGCC‐3′) (Gene Tools) in M2 medium containing 2.5 μM milrinone. To facilitate the degradation of protein, microinjected oocytes were maintained at GV arrest in M2 medium for 28 h, and then transferred to milrinone‐free M16 medium to resume the meiosis for further experiments.

### 2.3 cRNA construction and in vitro transcription

Wild‐type full‐length *Paxillin* and *Slk* (1‐373) cDNA were sub‐cloned into pCS2+/myc, pcDNA3.1/FLAG and pcDNA3.1/HA vectors, respectively. *Slk* (1‐373) *K63R* was sub‐cloned into pCS2+/myc. cRNA was synthesized from linearized plasmid using SP6 mMessage mMachine kit (ThermoFisher Scientific), and purified with Monarch® RNA cleanup kit (New England BioLabs). After micro‐injected with 10–15 pl of 500–1500 ng/μl cRNA, oocytes were arrested at the GV stage for 4 h for effective peptide synthesis, and then cultured in milrinone‐free M16 medium for further analysis.

### 2.4 Inhibitor treatment

Erlotinib (HY‐50896, MCE) and tubacin (HY‐13428, MCE) were dissolved in DMSO to 100 or 10 mM for stock solutions, which were further diluted in culture medium to a working concentration of 10 or 1 μM, respectively.

### 2.5 Immunofluorescence and microscopy

Oocytes were fixed in 2% paraformaldehyde (PFA) in PEM buffer (100 mM Pipes, pH 6.9, 1 mM MgCl_2_, 1 mM EGTA) with 0.5% Triton X‐100 for 1 h at room temperature, then washed and blocked for 1 h in phosphate buffer saline (PBS) added with 10% normal goat serum. The blocked oocytes were incubated in blocking buffer with primary antibodies at 4°C overnight. Antibodies used in the experiments are described in Table S1. After being washed three times (5 min each) in PBS containing 0.2% Triton X‐100, oocytes were labelled with appropriate secondary antibodies for 1 h at room temperature, then washed and mounted on glass slides in mountain medium with DAPI (Vector laboratories). The fluorescent signals from both control and experimental oocytes were acquired by setting up the same parameters of the upright fluorescent microscope (ZEISS Axio Imager A2) or confocal microscope(ZEISS 880 Airyscan) semi‐quantitative analysed by ImagePro Plus software and Zeiss analysis software.

### 2.6 Chromosome spreads

After the proper treatment, the oocytes were incubated momentarily in pre‐warmed acid Tyrode's solution (T1788, Sigma) to get rid of the encompassing zona pellucida. After a short recovery time in M2 medium, a group of 10 oocytes each time were carefully shifted to 100 μl drops of fixation solution (1% paraformaldehyde in distilled water with 0.1% Triton X‐100) on glass slides; these oocytes dilated and ruptured after a few seconds, and gradually “thawed” on the slides. The slides were air‐dried overnight. Before 1 h incubation in blocking solution, the slides were immersed in PBS to wash off any salt mixed in the chromosome samples. The chromosome samples were immune‐labelled with primary antibody at 4°C overnight. After washing in PBS, the samples were incubated with an appropriate secondary antibody for 1 h at room temperature and mounted in mountain medium with DAPI (Vector Laboratories) and observed under an upright fluorescent microscope (ZEISS Axio Imager A2).

### 2.7 Immunoblotting analysis

A total of 50–200 oocytes were lysed directly in Laemmli sample buffer (Bio‐Rad) and heated at 95°C for 10 min. The protein samples were separated on 10% SDS‐PAGE gel and transferred to polyvinylidene fluoride (PVDF) membrane (Millipore). To detect the phosphorylation levels of SLK, the samples were separated on 7.5% Mn^2+^‐Phos‐tag™ SDS‐PAGE (NARD) and transferred to the PVDF membrane. The membranes were blocked with tris‐buffered saline with Tween 20 (TBST), supplemented with 5% low fat dry milk for 1 h at room temperature and then incubated with the primary antibodies at 4°C overnight. Antibodies used in the experiments are described in Table S1. After washing three times in TBST, the membranes were incubated with horseradish peroxidase (HRP)‐conjugated secondary antibody and were detected with sensitive ECL solution (Vazyme) and the protein bands were visualized by Fusion Fx (Vilber Lourmat).

### 2.8 Cell culture and plasmid transfection

HEK‐293T cells were cultured in DMEM (Hyclone) containing 10% FBS (Gibco) and 1% penicillin–streptomycin solution (Gibco) at 37°C in 5% CO_2_ atmosphere. Plasmids were transfected into HEK‐293T cells using Lipofectamine 3000 (Invitrogen).

### 2.9 Immunoprecipitation and ubiquitination assay

At 48 h after transfection, cellular lysates were prepared by incubating the cells in lysis buffer (50 mM Tris–HCl [pH 7.5], 150 mM NaCl, 0.3% Nonidet P‐40, 2 mM EDTA) containing protease inhibitor cocktail (Roche) for 40 min at 4°C, followed by centrifugation at 14,000× g for 15 min at 4°C. According to the manufacturer's protocol, the protein concentration of the lysates was determined by a bicinchoninic acid (BCA) protein assay kit (Pierce). For immunoprecipitation, 500 μg of protein was incubated with 2 μg of specific antibodies for 12 h at 4°C with constant rotation, 50 μl 50% protein G agarose beads or 20 μl anti‐flag agarose beads were added and incubated for an additional 3 h at 4°C. Beads were then washed five times using the lysis buffer. Between washes, the beads were collected by centrifugation at 1000*×* g for 5 min at 4°C. The precipitated proteins were eluted from the beads by resuspending the beads in 2× SDS‐PAGE loading buffer and boiling for 10 min.

### 2.10 Proximity ligation assay

Proximity ligation assay (PLA) was performed using the Duolink® In situ Red Starter Kit Mouse/ Rabbit (Sigma‐Aldrich). According to the manufacturer's instructions, oocytes were pre‐processed with a sequence of fixation, recovery and permeabilization, similar with the immunofluorescence staining. The oocytes were blocked with Duolink® block buffer at 7°C for 1 h, and incubated with anti‐SLK and anti‐paxillin antibodies diluted in blocking solution, overnight at 4°C, then followed by 1‐h treatment in pre‐diluted anti‐rabbit plus and anti‐mouse minus probes at 37°C. Thereafter, the oocytes were consecutively incubated in 1× ligase and 1× polymerase, for 30 and 100 min, respectively, at 37°C, and then mounted on the slides with Duolink® In situ Mounting Medium with DAPI.

### 2.11 Statistical analysis

The data were expressed as the mean ± *SEM* of a minimum of three independent experimental replicates. Differences between treated groups were analysed by *t*‐test or one‐way ANOVA using GraphPad Prism 8.0 software (Hallogram Publishing, USA), and the level of significance was accepted as *P* < 0.05.

## 3 RESULT

### 3.1 SLK protein expression and subcellular localization during meiotic maturation in mouse oocytes

**FIGURE 1:**
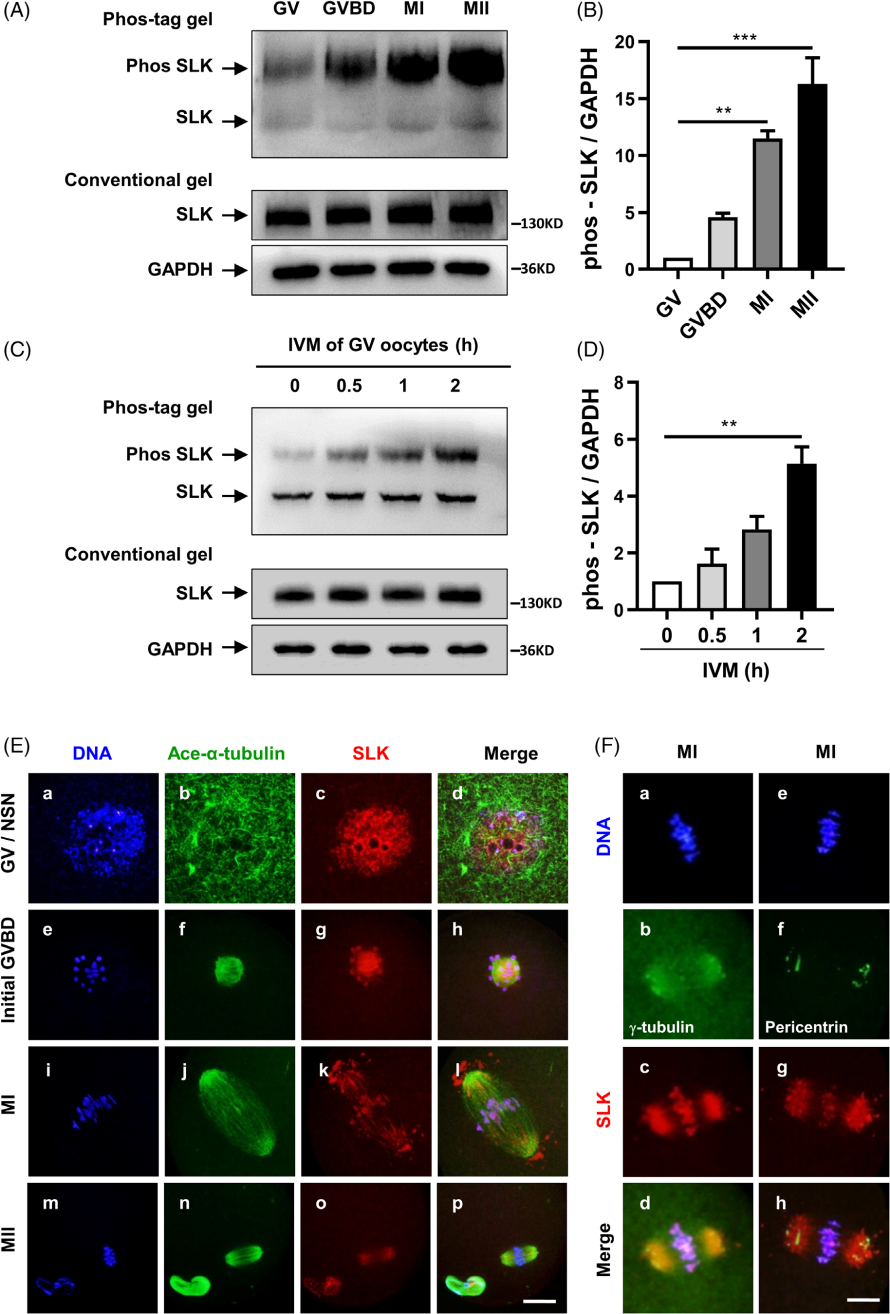
Expression and subcellular localization of Ste20‐like kinase (SLK) in mouse oocytes during meiosis. (A) The protein level and phosphorylated form of SLK were tested by conventional gel or Phos‐tag gel. Each sample contained 80 cells which were collected after 0, 2, 8 and 16 h in vitro maturation (IVM) culture, corresponding to germinal vesicle (GV), germinal vesicle breakdown (GVBD), metaphase I (MI), metaphase II (MII). (B) Quantitative analysis of phosphorylated SLK level at different meiotic stages. Data were presented as the mean percentage (mean ± *SEM*) of three independent experiments. **P < 0.01; ***P < 0.001, by ordinary one‐way ANOVA analysis. (C) The protein level and phosphorylated form of SLK were detected by conventional gel or Phos‐tag gel in oocytes around meiotic resumption. Each sample contained 80 cells collected after 0, 0.5, 1 and 2 h IVM. (D) Quantitative analysis of phosphorylated SLK level at special time points. Data were presented as the mean percentage (mean ± *SEM*) of three independent experiments. **P < 0.01 by ordinary one‐way ANOVA analysis. (E) Representative immunofluorescence images showing SLK subcellular localization of SLK in mouse oocytes at GV, GVBD, MI and MII. SLK was in red, Ace‐α‐tubulin in green and DNA in blue. Scale bar, 40 μm. (F) Representative immunofluorescence images showing the relationship between SLK (red) and γ‐tubulin (green) or Pericentrin (green), with DNA in blue, in MI oocytes. Scale bar, 10 μm

To investigate the role of SLK during meiotic maturation in mouse oocytes, its protein expression and subcellular location pattern were initially detected. As shown in Figure 1A, a single peptide band of SLK was detected by conventional western blot procedure in oocytes, which was at a high level and constant at different developmental stages during meiotic maturation. Especially an extra band, in significantly slow migration, was revealed using Phos‐tag™ acrylamide. Its signal was faint at the GV stage but markedly increased at GVBD and reached peak levels at MI and MII stages (Figure 1A,B). The level of SLK phosphorylation was 4.59, 11.48 and 16.29 folds higher at GVBD, MI and MII stages, respectively, compared with that at GV stage. For more details, the dynamics in SLK phosphorylation were further illustrated around the window phase from GV to GVBD during oocyte in vitro maturation culture with Phos‐tag™ acrylamide. SLK was barely phosphorylated at 0 h of culture, namely at the GV stage. Still, such modification was prominently increased after 0.5 h culture. At that time, oocytes theoretically entered the initial stage of GVBD, and the phosphorylation level went on increasing at the time point of 1 h culture. It rose to the highest at 2 h; at this moment, most oocytes have resumed meiosis in our culture system (Figure 1C,D). Based on the quantity analysis, the value of SLK phosphorylation was 1.62, 2.83 and 5.14 folds higher at 0.5, 1 and 2 h, respectively, compared with that at 0 h of culture. Obviously, SLK was detected as a single and stable band by conventional acrylamide gel, without any difference among the time points of 0, 0.5, 1 and 2 h. The result clearly shows that SLK undergoes dynamic phosphorylation modification around the meiotic resumption, suggesting its potential involvement in oocyte meiotic resumption and progression.

As revealed by immunofluorescence, SLK was mainly distributed in the nucleus at the GV stage, with no particular concentration in the cytoplasmic area (Figure 1E [a–d]). This kinase exhibited a completely different subcellular distribution upon meiotic resumption. It was localized to the condensing chromosomes and co‐localized with the reassembled microtubules around chromosomes after nuclear envelope breakdown (Figure 1E [e–h]). Along with the cell cycle to MI, SLK remained on chromosomes and overlapped with microtubules in the spindle apparatus; additionally, it was also distributed in a cytoplasmic area surrounding the spindle and beyond the spindle poles (Figure 1E [i–l]). Mainly, SLK was distributed as a uniform cloud in the spindle polar area, surrounding the highly condensed foci of γ tubulin (Figure 1F [a–d]) and pericentrin (Figure 1F [e–h]), two core components of microtubule organizing centres (MTOC). In MII oocytes, SLK was only labelled on the spindle, with no signal across chromosomes (Figure 1E [m–p]).

To reveal more detailed information about SLK distribution on chromosomes, immunofluorescence was conducted on chromosome spreads at different meiotic stages. As shown in Figure S1, the SLK signal was detected on the condensing chromosomes at early GVBD. It remained in a discontinuous bead distribution across chromosomes from GVBD to the MI stage (Figure S1E–L). However totally disappeared from chromatids at the MII stage (Figure S1M–P). Additionally, among all the stages, the SLK signal was always absent in the centromere region of chromosomes when co‐labelled with CREST serum. Such location pattern implies unique roles of SLK in the structural maintenance of homologous chromosomes during meiosis I.

### 3.2 Depletion of SLK impairs oocyte meiotic resumption

**FIGURE 2:**
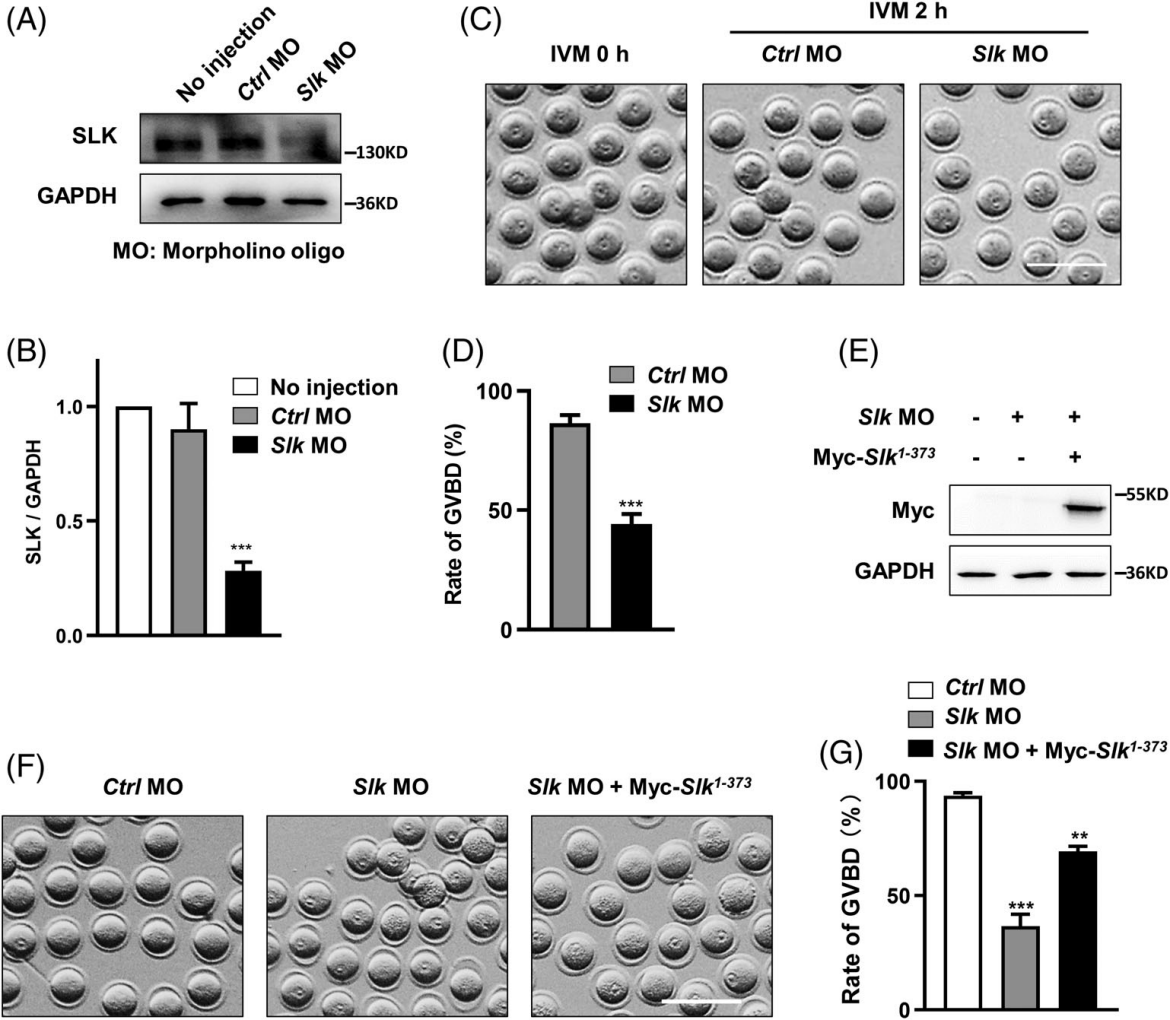
Depletion of Ste20‐like kinase (SLK) results in delayed meiotic resumption. (A) Western blot analysis of SLK protein in oocytes of the un‐injected group, control morpholino oligo (MO) group and *Slk* MO group. Each sample had 80 oocytes. (B) Quantitative analysis of SLK protein level in oocytes of control groups and *Slk* MO group. Data were presented as the mean percentage (mean ± *SEM*) of at least three independent experiments. ***P < 0.001 by ordinary one‐way ANOVA analysis. (C) Representative images of oocytes in control group and *Slk* MO group at 0 or 2 h of IVM, respectively. Scale bar, 200 μm. (D) Quantitative analysis of the GVBD rate in control group and *Slk* MO group after 2 h IVM. Data were presented as the mean percentage (mean ± *SEM*) of at least four independent experiments. Control group: *n =* 201 versus *Slk* MO group: *n =* 230. ***P < 0.001 by unpaired *t* test. (E) Myc‐protein level of oocytes with different microinjection treatments by western blot analysis. Each sample had 50 oocytes. (F) Representative images of oocytes in control group, *Slk* MO group and SLK rescue group (*Slk* MO + SLK^1‐373^) at 0 or 2 h of IVM, respectively. Scale bar, 200 μm. (G) Quantitative analysis of the GVBD rate in groups of control MO, *Slk* MO and *Slk* MO + SLK^1‐373^ after 2 h IVM. Data were presented as the mean percentage (mean ± *SEM*) of at least three independent experiments. Control group: *n =* 133 versus *Slk* MO group: *n =* 133 versus *Slk* MO + SLK^1‐373^ group: *n =* 103. ***P < 0.001; **P < 0.01 by ordinary one‐way ANOVA analysis

To explore SLK function at the window of meiotic resumption, tailored morpholino oligo microinjection was performed to block the translation of endogenous *Slk* mRNA in mouse oocytes. As shown in Figure 2, western blot and quantitative statistical analysis revealed SLK protein level was significantly decreased in *Slk* morpholino oligo group compared to control groups (Figure 2A,B). This also confirmed the specificity of the morpholino oligo sequence and the efficiency of our micro‐manipulation procedure. Further stereomicroscope observation and statistical analysis distinctly demonstrated that the rate of GVBD was dramatically reduced in oocytes with SLK knockdown (*Ctrl* MO = 86.46 ± 1.69 vs. *Slk* MO = 44.10 ± 2.14; Figure 2C,D), the decline tends of GVBD rate was reversed obviously when the exogenous mRNA of SLK kinase active fragment SLK^1‐373^ was co‐injected into oocytes with *Slk* morpholino sequence (*Ctrl* MO = 93.73 ± 1.25 vs. *Slk* MO = 36.63 ± 5.26 vs. *Slk* MO + Myc‐*Slk* ^1‐373^ cRNA = 69.37 ± 2.20; Figure 2E–H). This data also solidly eliminate any potential off‐target effects of *Slk* morpholino oligo used in our study. In line with the phenotype induced with oligo sequence, meiotic resumption was also effectively blocked in mouse oocytes by injection of mRNA of SLK kinase‐inactive mutant, SLK^1‐373; K63R^, as compared with the plasmid vehicle group (vehicle = 85.33 ± 1.71 vs. *Myc‐Slk* ^*1‐373; K63R*^ cRNA = 42.30 ± 1.67; Figure S2A). All these data definitely verified that SLK kinase activity is essential for oocytes to resume the meiotic maturation progression accurately.

In addition, the meiotic resumption was not declined in mouse oocytes after being treated for 2 h with 10 μM Erlotinib, a kinase inhibitor of SLK^26^ (DMSO = 87.20 ± 0.32 vs. 10 μM Erlotinib = 86.37 ± 1.27; Figure S2B), the discrepancy may be associated with the inappropriate concentration of this inhibitor used in the current study, or its targeting to SLK is not specific enough.

### 3.3 SLK promotes Plk1‐mediated activation of MPF

**FIGURE 3:**
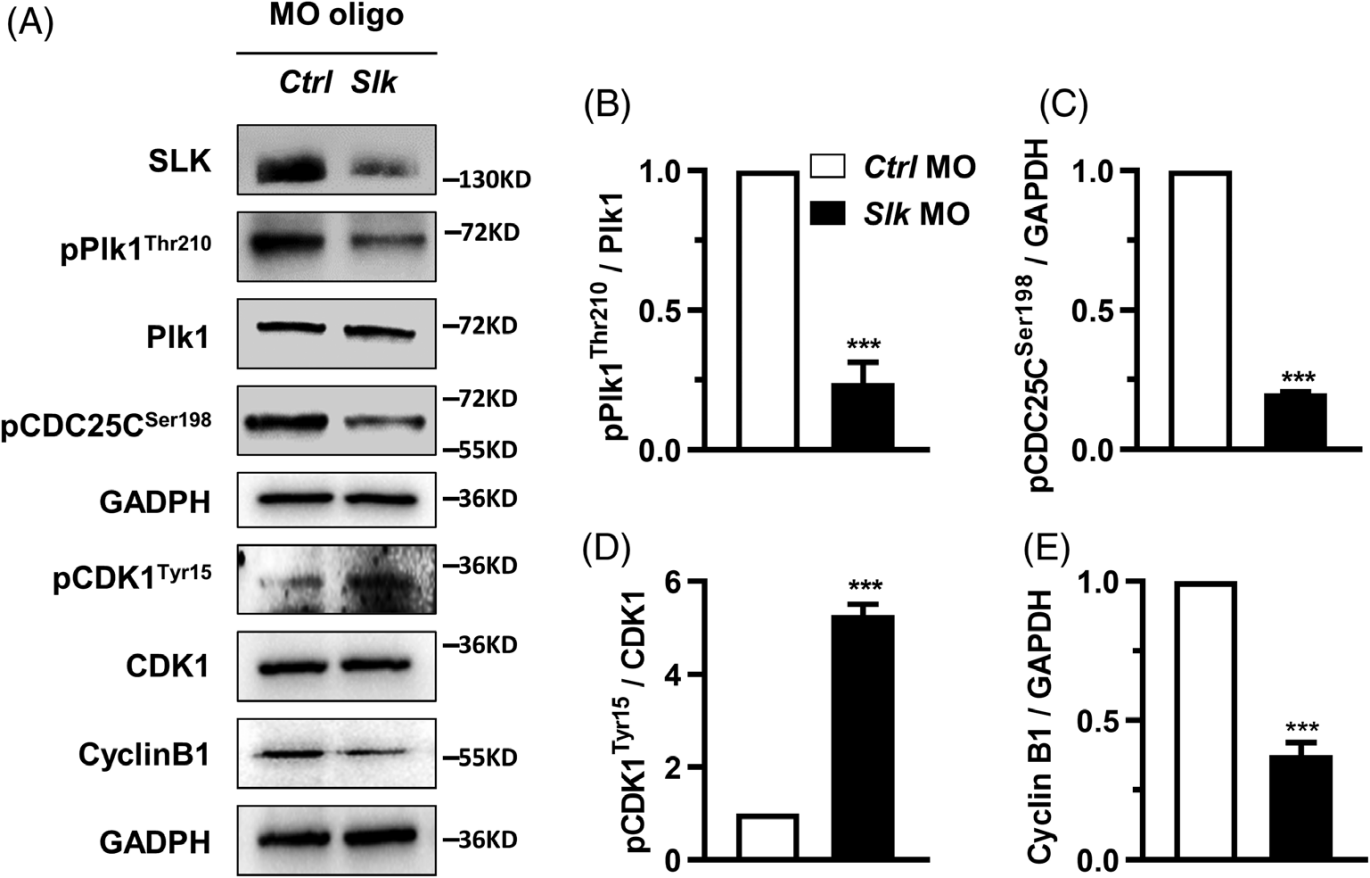
Ste20‐like kinase (SLK) depletion impairs metaphase‐promoting factor activity by down‐regulating Plk1‐CDC25C‐CDK1 cascade. (A) Changes in protein expression in oocytes in control and *Slk* morpholino oligo (MO) group by western blot. The blots were incubated with anti‐SLK, anti‐Plk1, anti pPlk1^Thr210^, anti‐pCDC25C^Ser198^, anti‐CDK1, anti CDK1^Tyr15^, anti‐Cyclin B1 and anti‐GAPDH antibodies, respectively. Each sample had 50–200 oocytes. (B–E) Quantitative analysis of protein level changes in oocytes. Data were presented as the mean percentage (mean ± *SEM*) of at least three independent experiments. ***P < 0.001 by unpaired *t* test

The meiotic resumption in female germ cells is similar to the G2/M transition in somatic mitosis, which requires the activity of Polo‐like kinase 1 (Plk1).^4^ It was previously reported that *Xenopus* polo‐like kinase Plx1 was pivotal for the meiotic resumption in *Xenopus* eggs, and its activation was stimulated by phosphorylation modification, which was catalysed by SLK.^21^ To explore the signal pathway inspired by SLK in mouse oocyte meiosis resumption, we first detected the level of Plk1 phosphorylation at Thr210 (pPlk1^Thr210^), a crucial site for Plk1 kinase activity, in oocytes with SLK depletion after 2 h maturation culture. As shown in Figure 3, the western blot analysis illustrated that the level of pPlk1^Thr210^ was significantly reduced in pace with SLK knockdown by morpholino oligo. Still, the gross Plk1 level was not affected (Figure 3A,B). Moreover, CDC25C Ser198 phosphorylation (pCDC25C^Ser198^), a specific downstream event of Plk1 activation,^27^ was prominently inhibited in SLK‐depleted oocytes (Figure 3A,C), which means the phosphatase activity of CDC25C targeting metaphase promoting factor (MPF) activation is suppressed. Consistent with this speculation, the inhibitory phosphorylation of cyclin‐dependent kinase 1 (CDK1), the catalytic subunit of MPF, was substantially higher in mouse oocytes injected with *Slk* morpholino oligo after 2 h culture (Figure 3A,D). These findings indicate that SLK may work to facilitate the activation and nuclear translocation of CDC25C, which then dephosphorylates CDK1 Tyr15, promoting MPF activation and, consequently, the resumption of meiotic progression in oocytes. Surprisingly, the accumulation of Cyclin B1, the regulatory subunit of MPF, was also reduced after SLK knockdown (Figure 3A,E), suggesting that SLK may be involved in additional molecular cascades governing MPF and meiosis proceeding in oocytes. The results above strongly support that the altered CDK1 activity and insufficient Cyclin B1 accumulation are the leading cause of GVBD delay in SLK‐depleted oocytes.

### 3.4 SLK participates in spindle assembly, chromosome alignment and kinetochore‐microtubule attachments in oocytes

**FIGURE 4:**
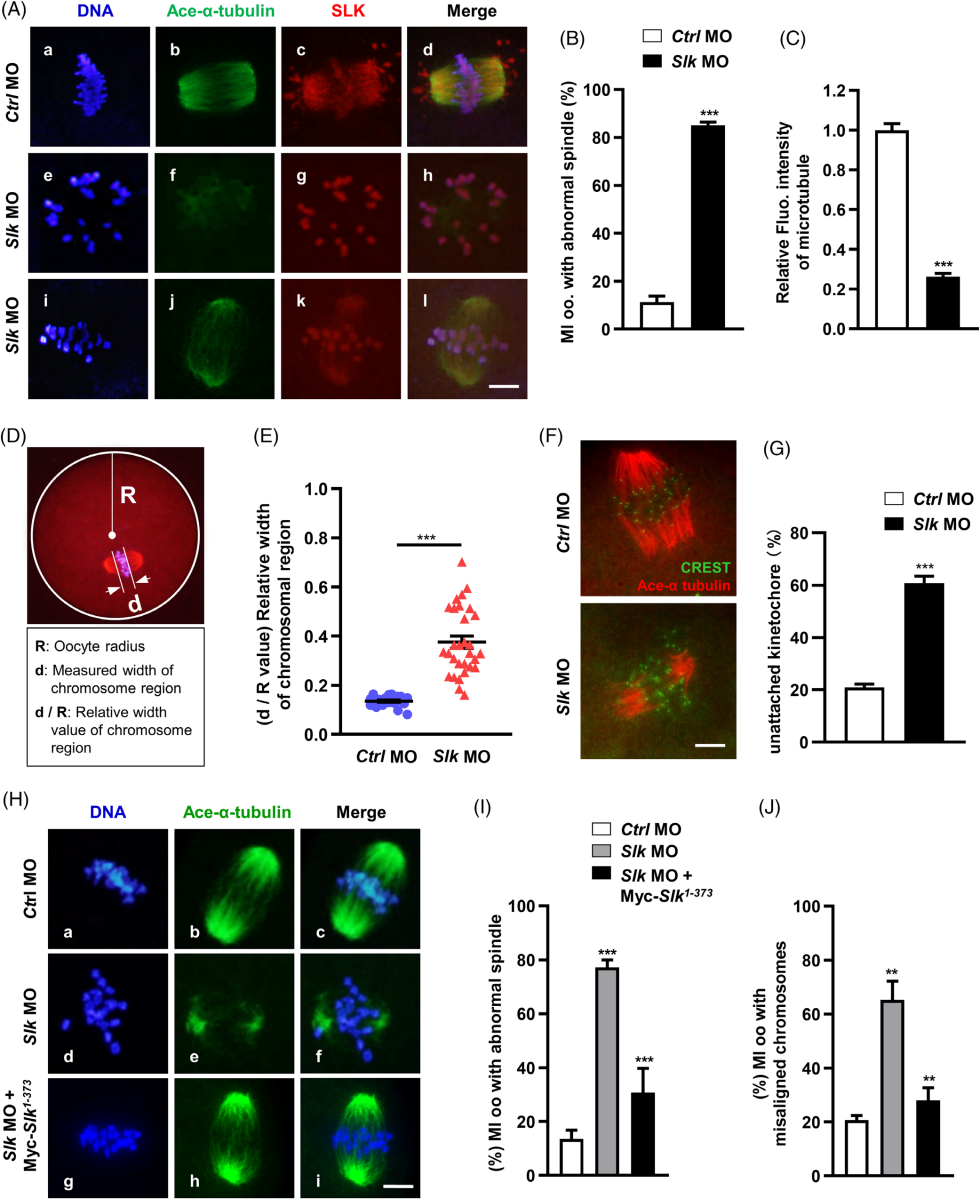
Ste20‐like kinase (SLK) depletion results in abnormalities in spindle assembly, chromosome alignment and K‐M attachment during oocyte meiotic maturation. (A) Representative images of spindle morphology and chromosome alignment in control and *Slk* morpholino oligo (MO) group. Oocytes were immunostained with anti‐acetylated‐α‐tubulin antibody, and then SLK antibody counterstained with DAPI to visualize chromosomes. Scale bar, 10 μm. (B) Quantitative analysis of the proportion of MI oocytes with abnormal spindles. Data were presented as the mean percentage (mean ± *SEM*) of at least three independent experiments. Control group: *n =* 163 versus *Slk* MO group: *n =* 134. ***P < 0.001 by unpaired *t* test. (C) Quantitative analysis of change in fluorescence intensity of acetylated‐α‐tubulin (Ace‐α‐tubulin). Data were presented as the mean percentage (mean ± *SEM*) of at least three independent experiments. Control group: *n =* 39 versus *Slk* MO group: *n =* 36. ***P < 0.001 by unpaired *t* test. (D) The calculation model of the relative chromosomal region width. The radius of the oocyte is represented as R; the width of the chromosome plate is represented as d. The relative chromosomal region width = d/R. (E) Quantitative analysis of the relative chromosomal region width. Data were presented as the mean percentage (mean ± *SEM*) of at least three independent experiments. Control group: *n =* 26 versus *Slk* MO group: *n =* 30. ***P < 0.001 by unpaired *t* test. (F) Representative images of kinetochore (K)–microtubule (MT) attachments in control and *Slk* MO oocytes. Scale bar, 5 μm. (G) Quantitative analysis of oocytes with unattached kinetochores. Data were presented as the mean percentage (mean ± *SEM*) of at least three independent experiments. Control group: *n =* 34 versus *Slk* MO group: *n =* 30. ***P < 0.001 by unpaired *t* test. (H) Representative images of spindle morphology in control and *Slk* MO and *Slk* MO + SLK^1‐373^ groups. Scale bar, 10 μm. (I) Quantitative analysis of MI oocytes with abnormal spindles. Data were presented as the mean percentage (mean ± *SEM*) of at least three independent experiments. Control group: *n =* 153 versus *Slk* MO group: *n =* 131 versus *Slk* MO + SLK^1‐373^ group: *n =* 137. ***P < 0.001 by ordinary one‐way ANOVA analysis. (J) Quantitative analysis of MI oocytes with misaligned chromosomes. Data were presented as the mean percentage (mean ± *SEM*) of at least three independent experiments. Control group: *n =* 143 versus *Slk* MO group: *n =* 164 versus *Slk* MO + SLK^1‐373^ group: *n =* 140. **P < 0.01 by ordinary one‐way ANOVA analysis.

As shown above, SLK localization on chromosomes and spindle apparatus implies its possible involvement in spindle assembly and maintenance. As expected, after 8 h maturation culture, the number of MI oocytes with abnormal spindle structure was significantly higher in *Slk* morpholino group than in groups of control morpholino and no‐injection (Figure 4A,B). The spindle abnormalities were manifested with defocused poles, non‐bipolar morphology, and, more frequently, extraordinarily low density of tubulin, as labelled with acetylated α‐tubulin (Figure 4A). Further fluorescence intensity analysis with ZEISS software confirmed the aggregating density of acetylated α‐tubulin was strikingly weak in SLK‐depleted oocytes (Figure 4C), indicating impaired stability of microtubules and definitely a kind of spindle with structural imperfections. The abnormal spindles were always companied with abnormal chromosome arrangement; statistical analysis confirmed an increased percentage of oocytes with misaligned chromosomes (*Ctrl* MO = 11.30 ± 2.52 vs. *Slk* MO = 85.13 ± 1.36; Figure 4A,B) and greater width of chromosome alignment (*Ctrl* MO = 0.14 ± 0.004 vs. *Slk* MO = 0.38 ± 0.025; Figure 4D,E) in *Slk* morpholino group, this may be due to unstable kinetochore‐microtubule attachment (K‐MT attachment), and thus a lacking of force driving chromosome movement and alignment. To verify this assumption, MI oocytes were immune‐stained with kinetochores and microtubules using CREST serum and anti‐acetylated‐α‐tubulin antibody after cold treatment, which must depolymerize the unstable microtubules that are not connected to kinetochores. Clearly, more dispersive kinetochores with few cold‐stable microtubules were observed in SLK‐depleted oocytes compared to control cells (*Ctrl* MO = 20.88 ± 1.31 vs. *Slk* MO = 60.75 ± 2.75; Figure 4F,G), such K‐MT attachment faults certainly lead to the unstable chromosomal bi‐orientation. In line with the analysis using morpholino oligo, identical defects in spindle morphology and chromosome alignment were also brought about when SLK activity was competitively inhibited by the kinase‐inactive mutant SLK^1‐373; K63R^ (vehicle = 17.80 ± 2.68 vs. *Myc‐Slk* ^*1‐373; K63R*^ cRNA = 67.93 ± 2.28; Figure S3A [a–f] and B), and additionally, such abnormalities were also copied by inhibiting SLK activity with 10 μM Erlotinib for 8 h (DMSO = 19.40 ± 1.19 vs. 10 μM Erlotinib = 55.00 ± 5.29; Figure S3A [g–l] and C). In favour of above data, co‐injection of mRNA of SLK kinase fragment SLK^1‐373^ partially rescued the defective spindle structure and chromosome arrangement in MI oocytes induced by *Slk* morpholino oligo (abnormal MI spindle: *Ctrl* MO = 13.50 ± 3.31 vs. *Slk* MO = 77.23 ± 2.72 vs. *Slk* MO + Myc‐*Slk* ^1‐373^ cRNA = 30.70 ± 5.26; misaligned chromosomes: *Ctrl* MO = 20.70 ± 1.62 vs. *Slk* MO = 65.27 ± 6.92 vs. *Slk* MO + Myc‐*Slk* ^1‐373^ cRNA = 27.97 ± 4.73; Figure 4H–J). Taken together, the evidence strongly supports that SLK activity is required for proper spindle assembly, chromosome alignment and kinetochore‐microtubule attachments in mouse oocyte meiotic progression.

### 3.5 MI arrest by spindle assembly checkpoint activity in SLK‐depleted oocytes

**FIGURE 5:**
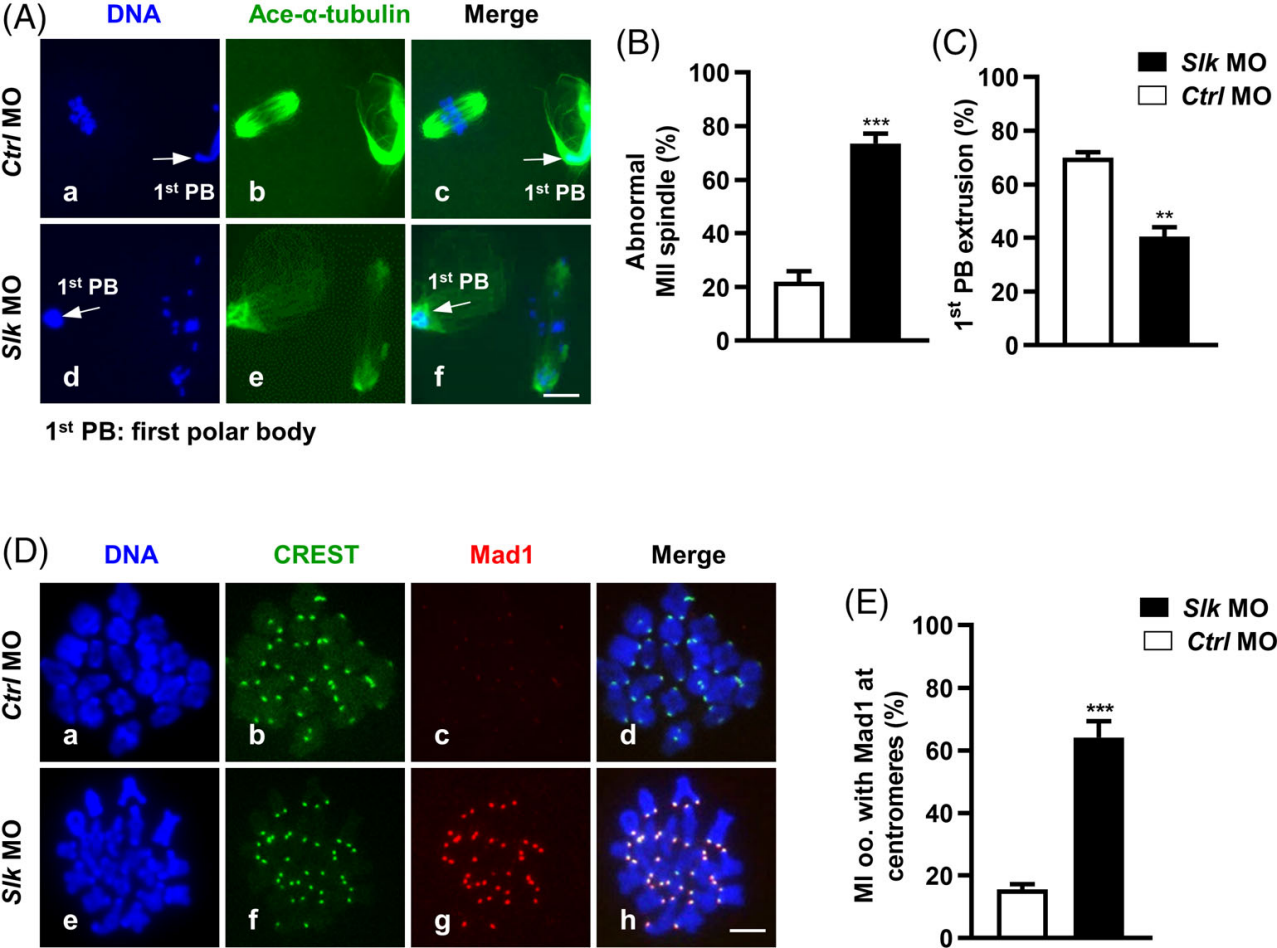
Ste20‐like kinase (SLK) depletion induces spindle assembly checkpoint activation and blocks 1st polar body (1st PB) extrusion. (A) Representative images of spindle morphology in MII oocytes. Oocytes were immunostained with anti‐acetylated‐α‐tubulin antibody and then counterstained with DAPI to visualize chromosomes. Scale bar, 10 μm. (B) Quantitative analysis of MII oocytes with abnormal spindles. Data were presented as the mean percentage (mean ± *SEM*) of at least three independent experiments. Control group: *n =* 66 versus *Slk* MO group: *n =* 50. ***P < 0.001 by unpaired *t* test. (C) Quantitative analysis of 1st PB1 extrusion rate. Data were presented as the mean percentage (mean ± *SEM*) of at least three independent experiments. Control group: *n =* 94 versus *Slk* MO group: *n =* 125. **P < 0.001 by unpaired *t* test. (D) Representative images of Mad1 signal in control and *Slk* MO group. Green: CREST; red: Mad1; blue: DNA. Scale bar, 2. 5 μm. (E) Quantitative analysis of MI oocytes with bright Mad1 signal at centromeres. Data were presented as the mean percentage (mean ± *SEM*) of at least three independent experiments. Control group: *n =* 71 versus *Slk* MO group: *n =* 58. ***P < 0.001 by unpaired *t* test

As shown in Figure 5, the number of oocytes that underwent first polar body (1st PB) extrusion was significantly reduced in oocytes with SLK knockdown after 16 h maturation culture (*Ctrl* MO = 70.03 ± 1.92 vs. *Slk* MO = 40.50 ± 3.52; Figure 5C), that means delayed meiotic transition from MI to MII in oocytes. Moreover, in SLK knockdown group, the majority of the MII oocytes contained abnormal spindles with misaligned chromosomes (*Ctrl* MO = 21.93 ± 3.93 vs. *Slk* MO = 73.53 ± 3.60; Figure 5A,B). The defects in spindle structure or its attachment with chromosomes cause chromosome congression failure and may provoke the activation of spindle assembly checkpoint (SAC) at centromeres, thus blocking anaphase onset and chromosome separation until all the errors are effectively corrected.^7^ To reveal the underlying mechanism arresting oocytes at the MI stage, the activity status of the SAC system was assessed in MI oocytes with SLK knockdown. As illustrated in Figure 5D,E, the fluorescence signal of SAC protein mitotic arrest deficient 1 (Mad1) at the centromere area was weak or not detected in control MI oocytes, but the bright Mad1 signal was labelled in the majority of SLK‐depleted oocytes (*Ctrl* MO = 15.60 ± 1.62 vs. *Slk* MO = 64.10 ± 5.21; Figure 5A,B), this indicates the persistent activation of SAC in oocytes, which may induce, at least partially, oocytes arrested at MI stage, and in logical consistence with the presence of defected spindle and chromosome alignment in oocytes without SLK activity.

### 3.6 SLK maintains α‐tubulin acetylation by regulating paxillin stability

**FIGURE 6:**
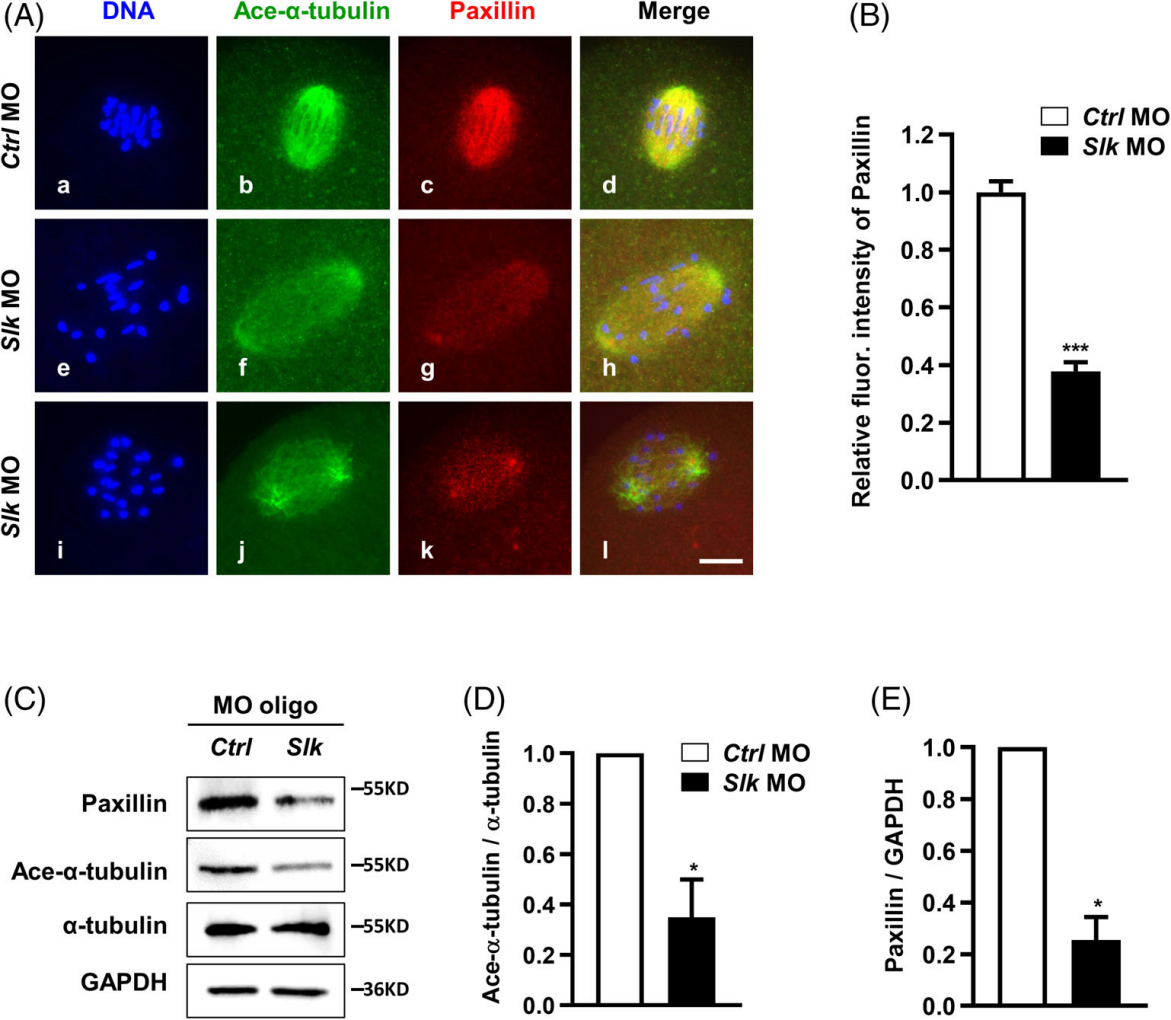
Ste20‐like kinase (SLK) depletion impairs α‐tubulin acetylation by down‐regulating paxillin. (A) Representative images of paxillin in oocytes in control and *Slk* morpholino oilgo (MO) group. Green: Ace‐α‐tubulin; red: paxillin; blue: DNA. Scale bar, 10 μm. (B) Quantitative analysis of paxillin fluorescence intensity. Data were presented as the mean percentage (mean ± *SEM*) of at least three independent experiments. Control group: *n =* 29 versus *Slk* MO group: *n =* 20. **P < 0.001 by unpaired *t* test. (C) Western blot analysis of proteins in oocytes after MO treatment. The blots were incubated with anti‐paxillin, anti‐α‐tubulin, anti‐Ace‐α‐tubulin and anti‐GAPDH antibodies, respectively. Each sample had 50–80 oocytes. (D) and (E) Quantitative analysis of changes in protein levels in oocytes. Data were presented as the mean percentage (mean ± *SEM*) of at least three independent experiments. *P < 0.05 by unpaired *t* test

In previous research, SLK is indicated to be co‐localized with paxillin, Rac1 and the microtubules at the leading edge of migrating cells,^28^ and paxillin promotes microtubule stability via modulating tubulin acetylation.^29^, ^30^, ^31^ The current study indicates that SLK was required for spindle assembly and was related to microtubule acetylation. To explore the underlying mechanism of SLK regulating microtubule stability, the protein expression and subcellular localization of paxillin were visualized and quantified by western blot and immunofluorescence staining. As shown in Figure 6, the fluorescent intensity of paxillin on the spindle was significantly decreased in SLK‐depleted oocytes (*Ctrl* MO = 1.00 ± 0.04 vs. *Slk* MO = 0.38 ± 0.03; Figure 6A,B); consistently, western blot confirmed that the protein level of paxillin was also apparently reduced as compared to controls (Figure 6C,E). In addition, western blot and quantitative measurement undoubtedly demonstrated the level of acetylated‐α‐tubulin was substantially decreased in *Slk* morpholino‐treated oocytes than in controls (Figure 6C,D); this reduction is strongly consistent with the phenotype illustrated by immunofluorescence in Figure 4A,C. Therefore, SLK seems to regulate tubulin acetylation through affecting paxillin expression.

To further confirm whether paxillin mediates SLK regulation on the spindle assembly and stability, exogenous paxillin was expressed in SLK‐depleted oocytes, and immunofluorescence was conducted in MI oocytes after 8 h maturation culture. As shown in the result, the percentages of aberrant spindle structure and misaligned chromosomes were remarkably decreased in oocytes co‐injected with *Slk* morpholino oligo and exogenous paxillin mRNA compared to oocytes only injected with *Slk* morpholino oligo (Figure S4A). The quantity analysis of fluorescence intensity illustrated that exogenous paxillin dramatically recovered the aggregation density of acetylated α‐tubulin on spindle (*Ctrl* MO = 1 ± 0.04 vs. *Slk* MO = 0.38 ± 0.04 vs. *Slk* MO + Myc‐*paxillin* cRNA = 0.99 ± 0.06; Figure S4B), this was further verified by western blot analysis (Figure S4C). Paxillin supplement also restored the relative width of chromosome alignment plate (*Ctrl* MO = 0.25 ± 0.01 vs. *Slk* MO = 0.41 ± 0.02 vs. *Slk* MO + Myc‐*paxillin* cRNA = 0.23 ± 0.01; Figure S4D). Taken together, paxillin functions as the downstream factor of SLK in modulating α‐tubulin acetylation‐mediated microtubule stability in mouse oocytes.

### 3.7 SLK protects paxillin from ubiquitylation‐mediated degradation through direct interaction

**FIGURE 7:**
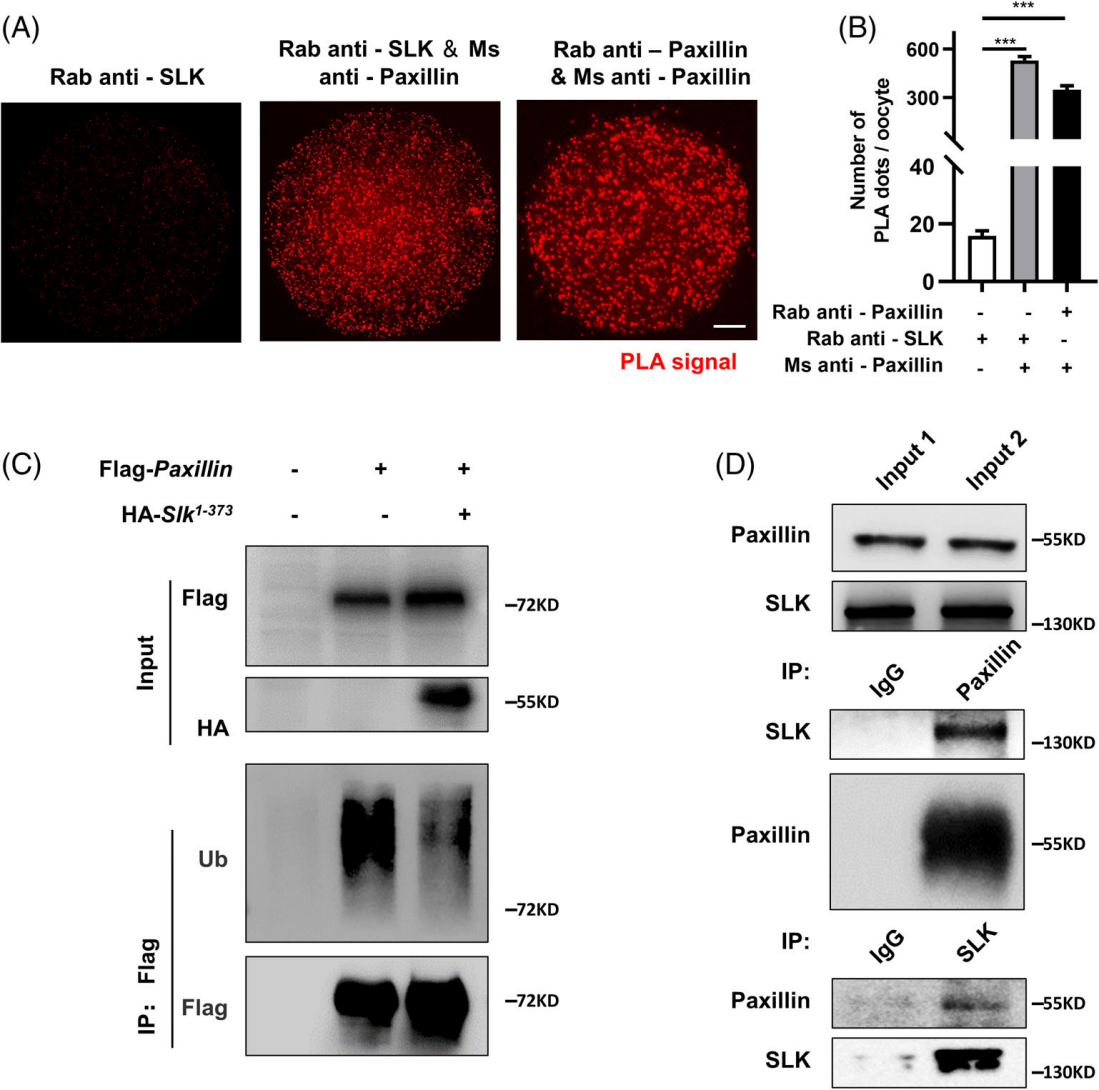
Ste20‐like kinase (SLK) prevents ubiquitylation‐mediated degradation of paxillin. (A) Representative images of PLA signals in different groups. Duolink‐PLA shows a direct interaction between SLK and paxillin in oocytes. Scale bar, 10 μm. (B) Quantitative analysis of PLA signal dots in different groups. >100 pixels dots are involved in the analysis. Data were presented as the mean percentage (mean ± *SEM*) of at least three independent experiments. Rabbit (Rab) anti‐SLK group: *n =* 21; Rab anti‐SLK & mouse (Ms) anti‐paxillin group: *n =* 21; Rab anti‐paxillin and ms anti‐paxillin group: *n =* 20. ***P < 0.001 by ordinary one‐way ANOVA analysis. (C) Co‐IP was performed to determine the interaction between SLK and paxillin. HEK 293T cell lysates were incubated with IgG/anti‐SLK antibody and IgG/anti‐paxillin antibody, respectively. The blots of IP eluates were probed with anti‐SLK and anti‐paxillin antibodies, respectively. (D) HEK 293T cells transiently transfected with plasmids encoding the indicated proteins were lysed and subjected to immunoprecipitation with an anti‐FLAG affinity gel. Input cell lysates and precipitates were immunoblotted with antibodies against FLAG and HA.

To confirm this hypothesis, the physical interaction between SLK and paxillin was first detected by the immunoprecipitation procedure. Duolink PLA analysis confirmed the direct physical interaction between SLK and paxillin in mouse oocytes (Figure 7A,B). Because of the limitation of proteins obtained from mouse oocytes, further immunoprecipitation assay was performed using somatic HEK‐293T cells, with paxillin antibody as bait to capture SLK. In line with the evidence from PLA analysis, the result showed that SLK was explicitly present in the cell lysate pre‐incubated with paxillin antibody instead of control IgG. Reciprocally, paxillin was also specifically captured by SLK antibody rather than no‐specific IgG (Figure 7D). In contrast to prior research, SLK depletion impacted the protein expression of paxillin, rather than its phosphorylation, in mouse oocytes. For further confirmation, paxillin and SLK^1‐373^ were overexpressed in HEK‐293T cells, respectively, and the level of paxillin ubiquitination was significantly decreased by SLK^1‐373^ overexpression (Figure 7C), suggesting SLK maintains paxillin stability by regulating its ubiquitination. Collectively, it is safe to assume that SLK benefits α‐tubulin acetylation and stability by suppressing paxillin ubiquitylation and degradation.

### 3.8 Tubacin inhibition of HDAC6 activity can restore microtubule stability in SLK‐depleted oocytes

**FIGURE 8:**
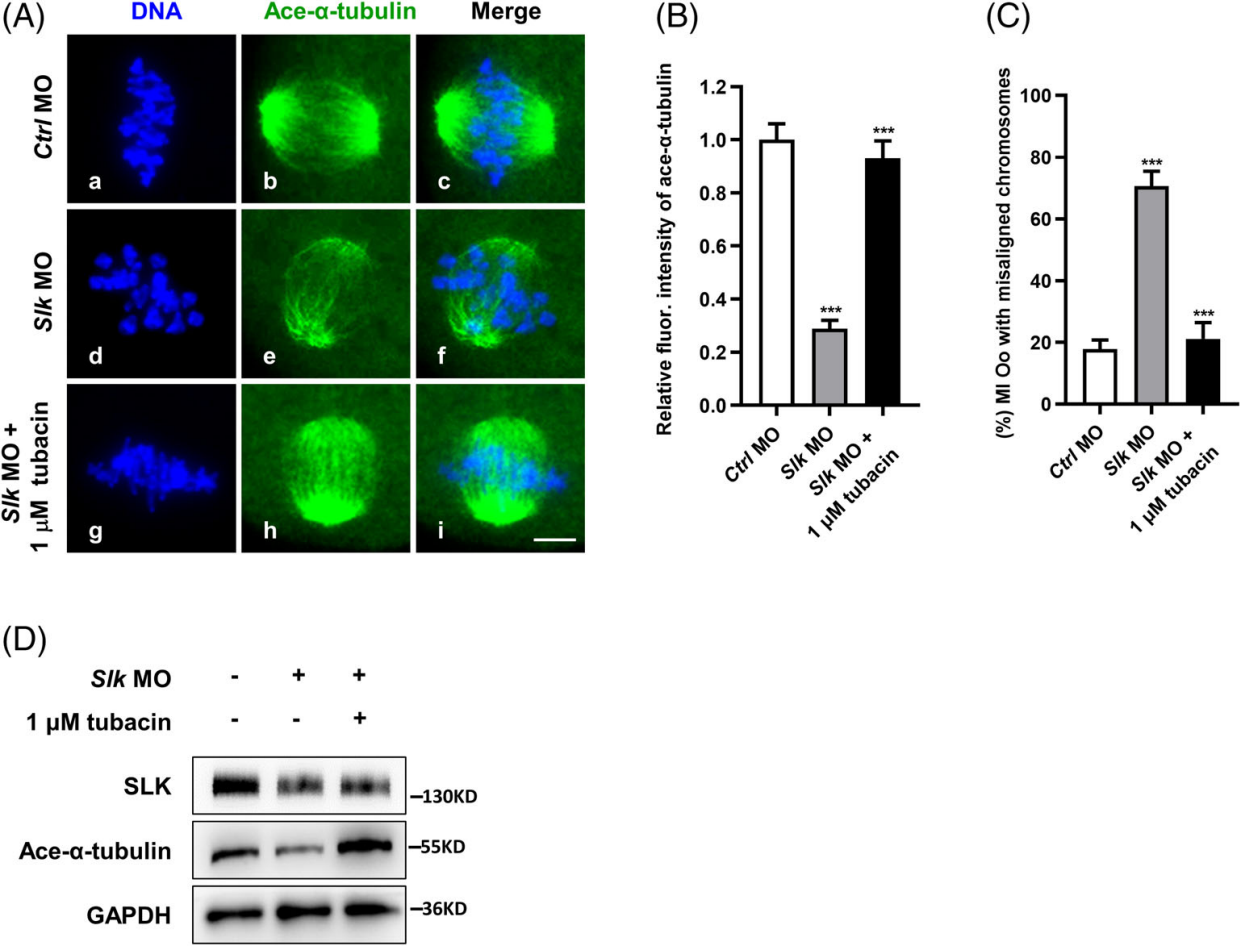
Pharmacological inhibition of HDAC6 restores microtubule stability in Ste20‐like kinase (SLK)‐depleted oocytes. (A) Representative images of spindle morphology and chromosome alignment in groups of control, *Slk* morpholino oilgo (MO) and *Slk* MO + 1 μM tubacin. Oocytes were immunostained with anti‐Acetylated‐α tubulin antibody and then counterstained with DAPI to visualize chromosomes. Scale bar, 10 μm. (B) Quantitative analysis of Ace‐α‐tubulin fluorescence intensity after treatments. Data were presented as the mean percentage (mean ± *SEM*) of at least three independent experiments. Control group: *n =* 26 versus *Slk* MO group: *n =* 15 versus *Slk* MO + 1 μM tubacin group: *n =* 15. ***P < 0.001 by ordinary one‐way ANOVA analysis. (C) Quantitative analysis of MI oocytes with misaligned chromosomes. Data were presented as the mean percentage (mean ± *SEM*) of at least three independent experiments. Control group: *n =* 50 versus *Slk* MO group: *n =* 33 versus *Slk* MO + 1 μM tubacin group: *n =* 36. ***P < 0.001 by ordinary one‐way ANOVA analysis. (D) Western blot analysis of protein levels of SLK and Ace‐α tubulin in control, *Slk* MO and *Slk* MO + 1 μM tubacin groups. The blots were incubated with anti‐Ace‐α‐tubulin, anti‐SLK and anti‐GAPDH antibodies, respectively. Each sample had 50–200 oocytes

It is known that paxillin works as the endogenous inhibitor against tubulin‐lysine deacetylase HDAC6, thus facilitating the rational level of α‐tubulin Lys40 acetylation and microtubule stability.^30^ To ask whether the balancing axis of paxillin‐HDAC6 mediates SLK regulation on α‐tubulin acetylation in oocyte meiotic spindle assembling, the situation of spindle and chromosome alignment was analysed when HDAC6 was inhibited with tubacin in SLK‐depleted oocytes. As shown in Figures 1 and 8 μM tubacin pronouncedly restored the defects in microtubule assembling and chromosome congression, as illustrated by quantitative analysis of ace‐α‐tubulin fluorescence intensity (*Ctrl* MO = 1 ± 0.06 vs. *Slk* MO = 0.29 ± 0.03 vs. *Slk* MO + 1 μM tubacin = 0.93 ± 0.07; Figure 8A,B) and proportion of dispersive chromosomes (*Ctrl* MO = 17.9 ± 2.9 vs. *Slk* MO = 70.7 ± 4.8 vs. *Slk* MO + 1 μM tubacin = 21.1 ± 5.3; Figure 8A,C). Furthermore, western blot analysis validated a more robust level of acetylated α‐tubulin in SLK‐depleted oocytes when HDAC6 activity was inhibited with tubacin in comparison with DMSO group (Figure 8D). All the results firmly support that SLK plays an important role in maintaining microtubule stability through regulating paxillin to suppress the deacetylation of α‐tubulin by HDAC6.

### 3.9 SLK reduction in aging oocytes and exogenous SLK contribution to meiotic progression and spindle assembly

**FIGURE 9:**
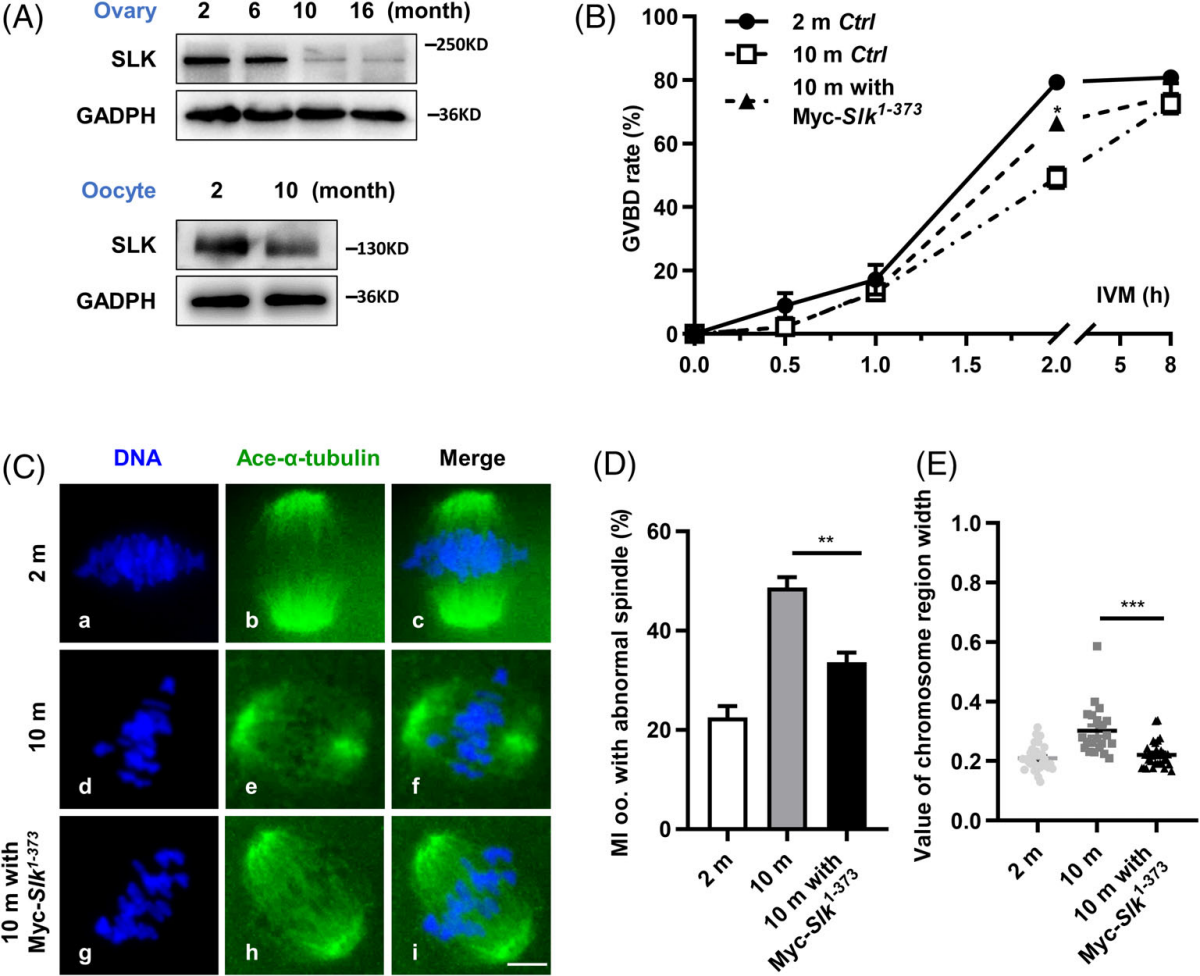
Ste20‐like kinase (SLK) reduction in aging oocytes and exogenous SLK contribution to meiotic progression and spindle assembly. (A) Western blot analysis of SLK levels in ovaries and oocytes of mice at different ages. The blots were incubated with anti‐SLK and anti‐GAPDH antibodies, respectively. Each sample had 60 oocytes. (B) Quantitative analysis of GVBD of oocytes after IVM with different treatments. Data were presented as the mean percentage (mean ± *SEM*) of at least three independent experiments. Young oocytes–oocytes from 2‐month‐old mice: *n =* 52; aging oocytes–oocytes from 10‐month‐old mice: *n =* 64; aging oocytes microinjected with Myc‐Slk^1‐373^: *n =* 50. *P < 0.05 by ordinary one‐way ANOVA analysis. (C) Representative images of spindle morphology and chromosome alignment in oocytes from different groups. Oocytes were immunostained with anti‐Ace‐α tubulin antibody and counterstained with DAPI. Scale bar, 10 μm. (D) Quantitative analysis of MI oocytes with the abnormal spindle in different groups. Data were presented as the mean percentage (mean ± *SEM*) of at least three independent experiments. Young (2 month) group: *n =* 70; aging (10 month) group: *n =* 89; aging oocytes microinjected with Myc‐Slk^1‐373^ group: *n =* 69. **P < 0.01 by ordinary one‐way ANOVA analysis. (E) Quantitative analysis of chromosomal region width in different groups. Data were presented as the mean percentage (mean ± *SEM*) of at least three independent experiments. Young group: *n =* 33; aging group: *n =* 21; aging with Myc‐Slk^1‐373^ group: *n =* 31. ***P < 0.001 by ordinary one‐way ANOVA analysis

Abnormal spindle assembly and chromosome separation errors are frequently observed in aging oocytes,^7^ but the molecular mechanism is still not fully understood. Here, by western blot analysis, we found the total protein level of SLK was dramatically reduced in ovary lysate from 10‐ and 16‐month‐old mice in comparison with 2‐ and 6‐month‐old mice; furthermore, in oocytes from 10‐month‐old mice, SLK expression was markedly decreased than that from 2‐month‐old mice (Figure 9A). Consistent with previous reports, these oocytes were manifested with delayed meiotic resumption and abnormal spindle structure, as well as misaligned chromosomes when analysed with immunofluorescence (Figure 9C), to a high extent, similar to abnormalities in SLK knocked down oocytes (Figure 4A), interestedly, these defects were effectively ameliorated by microinjection of exogenous SLK active fragment into aging oocytes (Figure 9B–E). Accordingly, the aging‐associated meiotic defects may be partially attributed to reduced SLK activity in oocytes.

## 4 DISCUSSION

This study thoroughly investigated SLK protein expression pattern and function in mouse oocytes during meiotic maturation. SLK is highly expressed in oocyte meiosis with an up‐regulated phosphorylation modification and multi‐site location on the nucleus, spindle and individualized chromosomes. SLK activity promotes the timely resumption of meiotic progression by inspiring the signal pathway of Plk1‐CDC25C‐CDK1. SLK also facilitates the acetylation of microtubules by maintaining paxillin level, so as to ensure correct spindle assembly and chromosome alignment in meiotic progression.

Current evidence demonstrates that SLK is ubiquitously expressed in murine adult tissues and most cell lines, but preferentially expressed in muscle and neuronal lineages in the developing embryo.^32^ Therefore constitutive SLK depletion led to early embryonic lethality in mice with significant developmental defects.^33^ Especially, muscle‐specific SLK depletion in adult mice displays a progressive myopathy, manifesting with a progressive increase in muscular weakness and fatigue.^34^ In mouse cortical neurons, loss of SLK leads to a less complex dendritic tree and impaired inhibition.^35^ In addition, podocyte‐specific deletion of SLK causes albuminuria in mice due to impaired cellular integrity.^36^ SLK autophosphorylation on the conserved sites Thr183, Ser189 and Thr193 within the activation segment, triggers a conformational change, promoting SLK activation and substrate binding,^19^, ^37^ and mutation of these residues results in a dramatic decrease in its kinase activity.^18^ SLK plays a critical role in cellular proliferation, migration and terminal differentiation through phosphorylating specific substrates, which have been identified so far, including Plk1, and cytoskeletal proteins RhoA, ezrin, paxillin and the p150 (Glued) dynactin subunit.^32^ It is still an ongoing investigation into the exact upstream mechanism stimulating SLK phosphorylation and activation.^19^

Up to the current date, SLK's role in mammalian germ cell development, particularly during meiotic progression in the testis and ovary, is still unresolved. Here, we found that SLK was expressed stably in mouse oocytes and phosphorylated around the resumption of meiosis; SLK knockdown with morpholino oligo or expression of kinase‐dead SLK K63R variant could substantially arrest oocytes at the GV stage. At the same time, the kinase active fragment could dramatically reverse the inhibitive effects of morpholino sequence, supporting the requirement of SLK activation for meiotic resumption in oocytes. This is consistent with an early report in *Xenopus* oocytes that SLK activity is essential for resuming meiotic progression. Still, the underlying mechanism and the molecular components of SLK‐dependent signalling pathways are not identified.^21^ In *Drosophila* S2 cells and fibroblasts, where the expression of a kinase‐inactive SLK mutant or SLK‐targeting siRNA also induces cell cycle arrest in early G2,^38^ and this has been proved to be attributed to the down‐regulated phosphorylation and activation of Plk1, an early trigger for G2/M transition.^20^, ^35^ Similarly, we revealed a significantly reduced level of Plk1 Thr210 phosphorylation in mouse oocytes with SLK depletion, indicating Plk1 may mediate SLK regulation on meiotic resumption. In somatic cells at G2/M transition, Plk1 activates the phosphatase CDC25C by phosphorylating Ser198 site, which then dephosphorylates Thr14 and Tyr15 in CDK1, and logically promoting full activation of MPF and, consequently, cell entering metaphase.^21^, ^39^ There is evidence that a kinase cascade comprised of xPlkk1 and Plx1, the *Xenopus* Plk homologue, triggers the activation of CDC25C during *Xenopus* oocyte maturation, but still short of factual data confirming a direct interaction between SLK and Plk1 signal cascade in oocyte context. Supporting this, we found that in SLK knockdown oocytes, reduced Plk1 phosphorylation is followed by decreased CDC25C phosphorylation and CDK1 dephosphorylation, as well as a weak accumulation of Cyclin B. The reduction in the above signal cascade doubtlessly cannot produce fully activated MPF, which logically accounts for the delayed onset of meiotic resumption in oocytes, at least partially.

However, some reports assume that CDC25C is dispensable for meiotic resumption in mouse oocytes unlike frog, instead CDC25B works as a dominant phosphatase responsible for CDK1 dephosphorylation and activation, also CDC25A plays similar role.^40^ In addition, Plk1 is not decisive for GVBD in mouse oocytes, unlike during somatic G2/M transition.^41^ Though SLK depletion delayed meiotic resumption and altered the phosphorylation status of Plk1, CDC25C and CDK1 in mouse oocytes, it is essential to clarify whether Plk1 is really the downstream target of SLK, and which isoform of CDC25 mediates SLK activity on MPF activation in mouse oocytes.

Previous evidence shows that Slik depletion in *Drosophila* S2 cells has been observed to result in various mitotic abnormalities, such as malformed astral microtubules and cells with off‐center spindles,^42^ while overexpression of active SLK is sufficient to induce ectopic spindle formation in human fibroblasts.^32^ Still, the underlying mechanism remains to be elucidated. In line with previous studies, we found that SLK is localized with the microtubule network in mouse oocytes during meiotic progression after GVBD, accompanied by the high phosphorylation level, and blocking SLK activity substantially destroyed meiotic spindle assembly and chromosome alignment, with decreased microtubule stability as the dominant phenotype. Given tubulin acetylation has been proven to strengthen the stability of microtubules and protect them against mechanical breakage,^43^, ^44^ we further confirmed that this modification is prominently reduced in SLK‐depleted oocytes and successfully rescued by co‐injection of human kinase active SLK fragment. So it is safe to say SLK promotes meiotic spindle assembly by regulating microtubule stability in oocytes. Recent evidence shows that SLK regulates focal adhesion assembly by targeting paxillin as the substrate,^45^, ^46^ and in polarized cell invasion and migration, this molecule works as an endogenous inhibitor against HDAC6, promoting microtubule acetylation.^30^ Coincidentally, we demonstrated that paxillin is co‐localized with microtubules in oocytes, and especially there is a direct physical interaction between paxillin and SLK in both mouse oocytes and somatic HEK 293T cells. SLK knockdown dramatically reduces paxillin expression, and the exogenous expression of paxillin could restore spindle defects and chromosome misalignment and decreased α‐tubulin acetylation in SLK‐depleted oocytes. We further found that SLK kinase activity can maintain paxillin at a reasonable level by suppressing its ubiquitination. These data demonstrate paxillin mediates the action of SLK in regulating microtubule stability in mouse oocytes. In addition, histone deacetylases HDAC8 and HDAC6 have been recently proved to negatively regulate α‐tubulin acetylation in mouse oocytes.^47^, ^48^ In logic consistence, we found that HDAC6 selective inhibitor tubacin can pronouncedly reverse spindle defects induced by paxillin reduction in SLK‐depleted oocytes. All the evidence supports that SLK facilitates α‐tubulin acetylation by preserving paxillin at a suitable level, thereby ensuring reasonable microtubule stability for the organizing and maintaining a functionally compete for spindle during oocyte meiotic division.

During meiotic progression in oocytes, chromosomes movement, alignment on the plate and separation to opposite poles are driven by force from spindle microtubules. As we found in SLK‐depleted oocytes, where chromosomes are not presented in a linear arrangement, but loosely aligned, even scattered in a pretty large area, such misaligning is primarily due to the unstable attachment of microtubules on the centromere area of chromosomes (Figure 4F). Theoretically, any deficiency in spindle structure or its attachment to chromosomes will spark the SAC system, which delays chromosome separation and anaphase onset until all these defects are appropriately rescued. In line with this logic, we found Mad1, a core component of SAC, is persisted on centromeres in SLK‐depleted MI oocytes, indicating the active SAC signal, which restrained meiotic transition to the MII stage, as we found only a small number of oocytes reach MII stages in SLK depletion group.

It is noteworthy that the protein level of SLK is prominently reduced in both ovaries and oocytes from aging mice; more interestedly, the exogenous SLK activity solidly benefits the meiotic progression and spindle assembly in aging oocytes. From a new perspective, this data strongly prove that SLK activity is vital for the punctual and accurate meiotic division process in oocytes and may be an objective of consideration for improving female reproductive disorders.

In addition, we found that SLK distributes across homologous chromosomes as discontinuous beads during meiosis I, but totally disappears from chromatids at MII stage, and always absents in centromere area at all stages. Not as expected, SLK signal is persistent across chromosomes in oocytes treated with *Slk* morpholino sequence, no matter weak or no SLK on the spindle area, thus it is inconvenient to evaluate SLK function here. SLK's location on chromosomes is different from synaptonemal complex (SC) and cohesins, which gather and form long strips along chromosome arms, but more like chiasmata, which establish connections between the homologues and help guide their proper bipolar alignment on the meiotic spindle.^49^, ^50^ When SC proteins disassemble from the chromosome arms at diplotene, the chromosomes are joined mainly by chiasmata,^49^ therefore the proper disjunction of homologous chromosomes requires the orderly resolution of chiasmata at anaphase I.^50^, ^51^ The mismatch repair (MMR) genes MLH1 and MLH3 are related to chiasmata structure and function, their dysfunction is highly associated with aneuploidy, pregnancy loss, and premature reproductive aging in human.^50^ Whether SLK is associated with chiasmata function in oocyte meiosis I remain to be explored in the future.

In conclusion, SLK functions are performed via diverse pathways during oocyte meiotic progression. These results provide insight into that, in oocyte meiotic resumption, SLK facilitates MPF maturation by activating the signal cascade involving Plk1, CDC25C and CDK1. SLK promotes the acetylation of microtubules by regulating paxillin expression to ensure correct spindle assembly and chromosome alignment in meiotic progression.

## AUTHOR CONTRIBUTIONS

Ke Song designed and performed experiments, collected, analysed and interpreted data; drafted the manuscript and participated in the preparation of its final version. Xiuying Jiang, Xiangning Xu, Ye Chen, Jiaqi Zhang and Ying Tian prepared experimental materials. Jing Weng, Qian Wang and Yuanjing Liang provided technical support and participated in the preparation of final version of manuscript. Wei Ma conceived and supervised the study, designed experiments, analysed, and interpreted data and was responsible for the final version of the manuscript.

## CONFLICT OF INTEREST

The authors declare no conflicts of interest.

## Supporting information


**Figure S1.** Localization of SLK across chromosomes at different meiotic stages. DNA in blue; CREST in green; SLK in red. Scale bar, 2.5 μm.
**Figure S2.** Effects of SLK kinase‐dead mutant and SLK inhibitor on GVBD in oocytes. (A) Quantitative analysis of GVBD rate in oocytes of vehicle group and *Myc‐Slk* ^*1‐373 K63R*^ cRNA group after 2 h IVM. Data were presented as the mean percentage (mean ± *SEM*) of at least three independent experiments. Plasmid vehicle group: *n* = 104 versus *Myc‐Slk* ^*1‐373; K63R*^ cRNA injection group: *n* = 110. ***P < 0.001 by unpaired *t* test. (B) Quantitative analysis of GVBD rate in groups of DMSO and 10 μM Erlotinib after 2 h IVM. Data were presented as the mean percentage (mean ± *SEM*) of at least three independent experiments. DMSO group: *n* = 125 versus 10 μM Erlotinib group: *n* = 133; *P* > 0.05 by unpaired *t* test.
**Figure S3.** Effects of SLK kinase‐dead mutant and SLK inhibitor on MI spindle morphology in oocytes. (A) Representative images of spindle morphology in different treatment groups. Oocytes were immunostained with anti‐acetylated‐α‐tubulin antibody and then counterstained with DAPI. Scale bar, 10 μm. (B) Quantitative analysis of MI oocytes with the abnormal spindle. Data were presented as the mean percentage (mean ± *SEM*) of at least three independent experiments. Plasmid vehicle group: *n* = 107 versus *Myc‐Slk* ^*1‐373; K63R*^ cRNA group: *n* = 95. ***P < 0.001 by unpaired *t* test. (C) Quantitative analysis of MI oocytes with the abnormal spindle. Data were presented as the mean percentage (mean ± *SEM*) of at least three independent experiments. DMSO group: *n* = 129 versus 10 μM Erlotinib group: *n* = 138. **P < 0.01 by unpaired *t* test.
**Figure S4.** Exogenous paxillin reverses defects in MI spindle structure and chromosome alignment in SLK‐depleted oocytes. (A) Representative images of spindle morphology and chromosome alignment in control, *Slk* MO and *Slk* MO + myc‐*paxillin* mRNA groups. Oocytes were immunostained with anti‐Acetylated‐α tubulin antibody and then counterstained with DAPI. Scale bar, 10 μm. (B) Quantitative analysis of Ace‐α‐tubulin fluorescence intensity in oocytes. Data were presented as the mean percentage (mean ± *SEM*) of at least three independent experiments. Control group: *n* = 38 versus *Slk* MO group: *n* = 32 versus *Slk* MO + myc‐*paxillin* mRNA group: *n* = 37. ***P < 0.001 by ordinary one‐way ANOVA analysis. (C) Western blot analysis of specific proteins in control, *Slk* MO and *Slk* MO + myc‐*paxillin* mRNA groups. The blots were incubated with anti‐myc, anti‐Ace‐α tubulin, anti‐SLK and anti‐GAPDH antibodies, respectively. Each sample had 50–200 oocytes. (D) Quantitative analysis of the relative value of chromosomal region width. Data were presented as the mean percentage (mean ± *SEM*) of at least three independent experiments. Control group: *n* = 27 versus *Slk* MO group: *n* = 34 versus *Slk* MO + myc‐*paxillin* mRNA group: *n* = 26. ***P < 0.001 by ordinary one‐way ANOVA analysis.
**Table S1.** The primary antibodies used in this study.

## Data Availability

The authors confirmed that all data needed to evaluate the conclusions in the paper are present in the paper and/or the Supplementary Materials. Additional data related to this paper may be requested from the authors.
