# Supplementary material for: Ste20‐like kinase activity promotes meiotic resumption and spindle microtubule stability in mouse oocytes
Source: Cell Prolif. 2022 Dec 29;56(4):e13391. doi: 10.1111/cpr.13391 (PMC10068952; doi:10.1111/cpr.13391)
Supplement: Supplementary file 1 — Figure S1. Localization of SLK across chromosomes at different meiotic stages. DNA in blue; CREST in green; SLK in red. Scale bar, 2.5 μm. Figure S2. Effects of SLK kinase‐dead mutant and SLK inhibitor on GVBD in oocytes. (A) Quantitative analysis of GVBD rate in oocytes of vehicle group and Myc‐Slk 1‐373 K63R cRNA group after 2 h IVM. Data were presented as the mean percentage (mean ± SEM) of at least three independent experiments. Plasmid vehicle group: n = 104 versus Myc‐Slk 1‐373; K63R cRNA injection group: n = 110. ***P < 0.001 by unpaired t test. (B) Quantitative analysis of GVBD rate in groups of DMSO and 10 μM Erlotinib after 2 h IVM. Data were presented as the mean percentage (mean ± SEM) of at least three independent experiments. DMSO group: n = 125 versus 10 μM Erlotinib group: n = 133; P > 0.05 by unpaired t test. Figure S3. Effects of SLK kinase‐dead mutant and SLK inhibitor on MI spindle morphology in oocytes. (A) Representative images of spindle morphology in different treatment groups. Oocytes were immunostained with anti‐acetylated‐α‐tubulin antibody and then counterstained with DAPI. Scale bar, 10 μm. (B) Quantitative analysis of MI oocytes with the abnormal spindle. Data were presented as the mean percentage (mean ± SEM) of at least three independent experiments. Plasmid vehicle group: n = 107 versus Myc‐Slk 1‐373; K63R cRNA group: n = 95. ***P < 0.001 by unpaired t test. (C) Quantitative analysis of MI oocytes with the abnormal spindle. Data were presented as the mean percentage (mean ± SEM) of at least three independent experiments. DMSO group: n = 129 versus 10 μM Erlotinib group: n = 138. **P < 0.01 by unpaired t test. Figure S4. Exogenous paxillin reverses defects in MI spindle structure and chromosome alignment in SLK‐depleted oocytes. (A) Representative images of spindle morphology and chromosome alignment in control, Slk MO and Slk MO + myc‐paxillin mRNA groups. Oocytes were immunostained with anti‐Acetylated‐α tubulin antibody and then co [file CPR-56-e13391-s001.docx]

**Ste20-like kinase (SLK) activity promotes meiotic resumption and spindle microtubule stability in mouse oocytes**

**Keywords:** SLK, GVBD, Spindle microtubule, Paxillin, Oocyte meiosis

Ke Song^2^, Xiuying Jiang^3^, Xiangning Xu^2^, Ye Chen^2^, Jiaqi Zhang^2^, Ying Tian^2^, Qian Wang^2^, Jing Weng^2^, Yuanjing Liang^2^, Wei Ma^1,2^

**^2^**Department of Histology and Embryology, School of Basic Medical Sciences, Capital Medical University, Beijing, 100069, China

^3^Division of Sport Anatomy, School of Sport Science, Beijing Sport University, Beijing, 100084, China

**^1^**Correspondences: Wei Ma, Department of Histology and Embryology, School of Basic Medical Sciences, Capital Medical University, 10 XiTouTiao, Youanmen, Beijing 100069, China, E-mail: mawei1026@ccmu.edu.cn

**Figure S1. Localization of SLK across chromosomes at different meiotic stages.** DNA in blue; CREST in green; SLK in red. Scale bar, 2.5 μm.

**Figure S2.** **Effects of SLK kinase-dead mutant and SLK inhibitor on GVBD in oocytes.** **(A)** Quantitative analysis of GVBD rate in oocytes of vehicle group and *Myc-Slk^1-373; K63R^* cRNA group after 2 h IVM. Data were presented as the mean percentage (mean ± SEM) of at least three independent experiments. Plasmid vehicle group: n = 104 vs. *Myc-Slk^1-373;K63R^* cRNA injection group: n = 110. *** P < 0.001 by unpaired *t* test. **(B)** Quantitative analysis of GVBD rate in groups of DMSO and 10 μM Erlotinib after 2 h IVM. Data were presented as the mean percentage (mean ± SEM) of at least three independent experiments. DMSO group: n = 125 vs. 10 μM Erlotinib group: n = 133; P > 0.05 by unpaired *t* test.

**Figure S3.** **Effects of SLK kinase-dead mutant and SLK inhibitor on MI spindle morphology in oocytes.** **(A)** Representative images of spindle morphology in different treatment groups. Oocytes were immunostained with anti-Acetylated-α-tubulin antibody and then counterstained with DAPI. Scale bar, 10 μm. **(B)** Quantitative analysis of MI oocytes with the abnormal spindle. Data were presented as the mean percentage (mean ± SEM) of at least three independent experiments. Plasmid vehicle group: n = 107 vs. *Myc-Slk^1-373;K63R^* cRNA group: n = 95. *** P < 0.001 by unpaired *t* test. **(C)** Quantitative analysis of MI oocytes with the abnormal spindle. Data were presented as the mean percentage (mean ± SEM) of at least three independent experiments. DMSO group: n = 129 vs. 10 μM Erlotinib group: n = 138. ** P < 0.01 by unpaired *t* test.

**Figure S4.** **Exogenous Paxillin reverses defects in MI spindle structure and chromosome alignment in SLK - depleted oocytes. (A)** Representative images of spindle morphology and chromosome alignment in control, *Slk* MO and *Slk* MO + myc-*Paxillin* mRNA groups. Oocytes were immunostained with anti-Acetylated-α tubulin antibody and then counterstained with DAPI. Scale bar, 10 μm. **(B)** Quantitative analysis of Ace-α-tubulin fluorescence intensity in oocytes. Data were presented as the mean percentage (mean ± SEM) of at least three independent experiments. Control group: n = 38 vs. *Slk* MO group: n = 32 vs. *Slk* MO + myc-*Paxillin* mRNA group: n = 37. *** P < 0.001 by ordinary one-way ANOVA analysis. **(C)** Western blot analysis of specific proteins in control, *Slk* MO and *Slk* MO + myc-*Paxillin* mRNA groups. The blots were incubated with anti- myc, anti- Ace-α tubulin, anti-SLK and anti- GAPDH antibodies, respectively. Each sample had 50-200 oocytes. **(D)** Quantitative analysis of the relative value of chromosomal region width. Data were presented as the mean percentage (mean ± SEM) of at least three independent experiments. Control group: n = 27 vs. *Slk* MO group: n = 34 vs. *Slk* MO + myc-*Paxillin* mRNA group: n = 26. *** P < 0.001 by ordinary one-way ANOVA analysis.

| **Table S1. The primary antibodies used in this study** | | | |
| --- | --- | --- | --- |
| **Antibody** | **Manufacturer** | **Cat^#^** | **Application** |
| HA Tag Monoclonal antibody | Proteintech | 66006-2-Ig | WB 1:2000 |
| DYKDDDDK tag Recombinant antibody (Binds to FLAG® tag epitope) | Proteintech | 80010-1-RR | WB 1:5000 |
| CDK1-Specific Polyclonal antibody | Proteintech | 19532-1-AP | WB 1:500 |
| ubiquitin Polyclonal antibody | Proteintech | 10201-2-AP | WB 1:500 |
| Purified Mouse Anti-Mouse Pericentrin | BD biosciences | 611814 | IF 1:50 |
| Anti-γ-Tubulin antibody, Mouse monoclonal | Sigma-Aldrich | T5326 | IF 1:100 |
| Anti-Acetylated Tubulin antibody, Mouse monoclonal | Sigma-Aldrich | T7451 | IF 1:5000; WB 1:1000 |
| Anti-GAPDH antibody produced in rabbit | Sigma-Aldrich | G9545 | WB 1:6000 |
| Human centromere auto serum(CREST) | Fitzgerald | 90C-CS1058 | IF 1:100 |
| Phospho-CDC25C (Ser198) Antibody | Affinity biosciences | AF4417 | WB 1:500 |
| Anti-paxillin Antibody (B-2) | Santa Cruz Biotechnology | sc-365379 | IF 1:250; WB 1:1000 |
| Anti-Mad 1 Antibody | Santa Cruz Biotechnology | SC-137025 | IF 1:25 |
| Anti-c-Myc Mouse Monoclonal Antibody | TransGen Biotech | HT101-01 | WB 1:500 |
| Cyclin B1 Antibody | Cell Signaling Technology | 4138 | WB 1:500 |
| Phospho-cdc2 (Tyr15) (10A11) Rabbit mAb | Cell Signaling Technology | 4539 | WB 1:500 |
| SLK antibody | GeneTex | GTX131438 | WB 1:1000; IF 1:500; PLA 1:500 |
| PLK1 antibody [N2C2], Internal | GeneTex | GTX104302 | WB 1:1000 |
| Recombinant Anti-PLK1 (phospho T210) antibody | Abcam | ab155095 | WB 1:500 |
| Paxillin Polyclonal Antibody | ImmunoWay Biotechnology | YT3606 | PLA 1:100 |
|  |  |  |  |
